# Control effect of root exudates from mycorrhizal watermelon seedlings on Fusarium wilt and the bacterial community in continuously cropped soil

**DOI:** 10.3389/fpls.2023.1225897

**Published:** 2023-09-11

**Authors:** Wei Li, Xue-Yi Hu, Cheng-Shang Zhu, Shao Xia Guo, Min Li

**Affiliations:** ^1^ College of Landscape Architecture and Forestry, Qingdao Agricultural University, Qingdao, Shandong, China; ^2^ Institute of Mycorrhizal Biotechnology, Qingdao Agricultural University, Qingdao, Shandong, China

**Keywords:** arbuscular mycorrhizal fungi, watermelon, root exudates, Fusarium wilt, bacterial community

## Abstract

Watermelon (*Citrullus lanatus*) is susceptible to wilt disease caused by *Fusarium oxysporum* f. sp *niveum* (FON). AMF colonization alleviates watermelon wilt and regulates the composition of root exudates, but the effects of mycorrhizal watermelon root exudates on watermelon Fusarium wilt is not well understood. Root exudates of watermelon inoculated with AMF (*Funeliformis mosseae* or *Glomus versiformme*) were collected in this study. Then the root exudates of control plants and mycorrhizal plants were used to irrigate watermelon in continuous cropping soil, respectively. Meanwhile, the watermelon growth, antioxidant enzyme activity, rhizosphere soil enzyme activities and bacterial community composition, as well as the control effect on FON were analyzed. The results indicated that mycorrhizal watermelon root exudates promoted the growth of watermelon seedlings and increased soil enzyme activities, actinomyces, and the quantity of bacteria in rhizosphere soil. The proportion of Proteobacteria and Bacteroides was decreased, and the proportion of Actinobacteria, Firmicutes, and Chloroflexi in rhizosphere soil was increased when the seedlings were watered with high concentrations of mycorrhizal root exudates. The dominant bacterial genera in rhizosphere soil were *Kaistobacter*, *Rhodanobacter*, *Thermomonas*, *Devosia*, and *Bacillus*. The root exudates of mycorrhizal watermelon could reduce the disease index of Fusarium wilt by 6.7–30%, and five ml/L of watermelon root exudates inoculated with *F. mosseae* had the strongest inhibitory effect on watermelon Fusarium wilt. Our results suggest mycorrhizal watermelon root exudates changed the composition of bacteria and soil enzyme activities in rhizosphere soil, which increase the resistance of watermelon to Fusarium wilt and promoted the growth of plants in continuous cropping soil.

## Introduction

Watermelon (*Citrullus lanatus*) is an annual or perennial vine that originated in Africa. Watermelon has become an important fruit in China for its strong adaptability, short cultivation cycle, and large market demand. According to statistics from the National Western Melon Industrial Technology System, the watermelon planting area in China is 1.533–1.667 million hm^2^, making China the world’s largest watermelon producer and consumer (Ministry of Agriculture, PRC, 2018). However, in modern intensive agricultural systems, continuous cropping is a common approach for cultivating watermelon to maximize yield and economic benefits. The incidence of diseases and pests has increased, and this has resulted in decreases in plant growth and development as well as yield and quality during the continuous cropping of watermelon (Wu et al., 2017). Fusarium wilt is a globally significant disease of watermelon. The pathogenic fungus that causes Fusarium wilt is *Fusarium oxysporum* f. sp. *niveum* (FON), which infiltrates the plant through a root wound or intercellular space at the top of a root hair and produces toxins that destroy cells and block the vascular bundles, thus affecting water transport and eventually causing wilting and death from dehydration (Jiang et al., 2019). This disease has become one of the main factors limiting watermelon production (Miller et al., 2020).

Many methods have been used to control watermelon Fusarium wilt, outing disease-resistance breeding (Hawkins et al., 2001), chemical control (Fravel et al., 2005), and grafting, soil disinfection, and biological control (Blanco et al., 2007). Biological control uses antagonistic microorganisms to inhibit the proliferation of pathogens. Therefore, the watermelon rhizosphere soil environment and soil microbial structure rapidly increase after inoculating watermelon plants with beneficial microorganisms. This approach can be used to overcome some of the challenges associated with the continuous cropping of watermelon (Xue et al., 2019).

Some soil microorganisms play important roles in inhibiting soil-borne pathogens and promoting the health of crops (Kennedy and Smith, 1995). Arbuscular mycorrhizal fungi (AMF), which are widely distributed in nature, form symbiotic relationships with approximately 71% of vascular plants (Fernández et al., 2019). After AMF invade the cortical cells of the host root system, they promote the absorption of large amounts of trace elements by host plants, particularly phosphorus. In return, the host plant provides photosynthates for the AMF (Bonfante and Genre, 2010). In addition, AMF play a significant role in preventing and controlling soil-borne diseases (Hage-Ahmed et al., 2013; Lewandowski et al., 2013). Many studies have shown that AMF help the host resist *Thielaviopsis basicola* (Schonbeck and Dehne, 1977), *Sclerotium cepivorum* (Torres-Barragán et al., 1996), *Fusarium oxysporum* (Hao et al., 2005), and others with high efficacy. Inoculation with AMF significantly reduces the amount of FON in the rhizosphere soil of watermelon, thus reducing the incidence of Fusarium wilt (Ren et al., 2015).

Inoculation with AMF impedes the invasion of pathogens by altering soil enzyme activities, promoting the enzyme defense system, affecting the composition of root exudates, and changing the rhizosphere microbial community. As an active component in the soil, soil enzymes directly participate in important biochemical processes, and their activities can be used as indexes for evaluating soil productivity and ecological health (Ndiaye et al., 2000). Zhao et al. (2010) reported that the activities of soil protease, urease, and polyphenol oxidase in watermelon cultivated under continuous cropping decrease with the number of continuous cropping years. However, after AMF form mycorrhizae with plants, soil enzyme activities and soil fertility increase significantly (Jia et al., 2020).

Bacteria produce large amounts of reactive oxygen species (ROS) when they infect plants, which induces damage. Superoxide anion free radicals are removed by superoxide dismutase (SOD), peroxidase (POD), and catalase (CAT), which decompose hydrogen peroxide (H_2_O_2_) into water (H_2_O) and oxygen (O_2_) (Asada, 2006; Huseynova et al., 2014; Shafi et al., 2015). The root activities of chitinase, β-1, 3-glucanase, phenylalanine ammonia lyase, CAT, and POD increased significantly after the formation of arbuscular mycorrhizae, and the resistance to stress increases in grafted or self-rooted watermelon seedlings (Chen et al., 2013).

The root system releases chemicals to promote or inhibit the production of pathogens (Dixon, 2001; D’Auria and Gershenzon, 2005). Hao et al. (2010) showed that after infecting watermelon with FON, allelopathic compounds, such as phthalic acid, salicylic acid, *p*-hydroxybenzoic acid, and ferulic acid, were detected in the root exudates. Small changes in the composition and content of the root exudates can lead to large changes in the composition of the rhizosphere microbial community (Badri and Vivanco, 2009; Ling et al., 2011). The secondary metabolic products hydroxybenzoic acid and rutin increase significantly after *Rhizophagus irregularis* infects *Viola triclor* (Zubek et al., 2015). AMF also affect the composition of rhizosphere microorganisms, thereby reducing the number of pathogens (Larsen and Jakobsen, 2003). In conclusion, improvements in host disease resistance by AMF are complex and may have local and systemic effects (Pozo and Azcón-Aguilar, 2007).

Previous studies have shown that AMF inoculation can promote watermelon growth and inhibit *F. oxysporum* infection (Ren et al., 2015). Root exudates have effects on soil physical properties and rhizosphere microbial communities (Paterson et al., 2007), both of which have positive or negative effects on soil-borne diseases. The composition of plant root exudates also changes according to biotic and abiotic environments (Bais et al., 2006). However, whether the root exudates of mycorrhizal plants can produce the same inhibitory effect as those inoculated with AMF remains unclear. Comparative studies of the inhibitory effects of plant root exudates inoculated with different AMF are needed. The objectives of this study were to (1) compare the effects of root exudates from watermelon inoculated with and without AMF on watermelon growth and the antioxidant enzyme system; (2) study the control effects of different root exudates on watermelon Fusarium wilt; and (3) determine the effects of different root exudates on soil enzyme activities and the bacterial community composition of watermelon. The results of this study provide new insights that will aid the control of watermelon Fusarium wilt by AMF.

## Materials and methods

### Experimental materials

The experiments were conducted in Qingdao, Shandong Province 36°16’N, 20°24’E, 9 m above sea level, China. The region has a warm temperate monsoon climate and brown soil. The mean annual precipitation is approximately 662 mm, the annual average temperature is 12.7°C, and the annual average relative humidity of 73%. The experiment was carried out in the solar greenhouse of Qingdao Agricultural University. The watermelon variety ‘Jingxin 4’ was provided by Beijing Jingyan Yinong Science and Technology Development Center (Beijing, China). *Funneliformis mosseae* and *Glomus versiforme* (provided by the Mycorrhizal Biotechnology Institute of Qingdao Agricultural University) were used in the experiment. The fungus was an inoculation mixture composed of mycorrhizal root segments and mycelia in soil and rhizosphere soil after propagating clover in sterilized sandy soil for 4–5 months. The XAD-4 macroporous adsorbent resin was purchased from Sigma-Aldrich Co. (St. Louis, MO, USA). It was washed with running hot water and then extracted with acetone, acetonitrile, and ether for 24 hours (Tang and Young, 1982). The cleaned resin was stored in a dark glass container with methanol until use.

### Collection of root exudates

The watermelon seeds were first washed and soaked in the water bath at constant temperature (55°C) for 30 min; the seeds were then incubated at 28–30 °C before seeding in plastic pots (16 cm × 16 cm height and diameter) containing 0.5 kg of soil medium. The physicochemical properties of the soil medium were as follows: total organic matter = 8.6 g·kg^-1^; available nitrogen = 11.2 mg·kg^-1^; available phosphorus = 19.1 mg·kg^-1^; exchangeable potassium = 10.3 mg·kg^-1^; and pH 7.10 (1:2, soil: water ratio).

Watermelon seedlings were divided into three equal groups and inoculated with *F. mosseae* (M), *G. versiforme* (V), and sterilized inoculant (C). There were three replicates per treatment, and three seedlings per replicate were retained. For inoculation with *F. mosseae* or *G. versiforme*, the growth medium was inoculated with 50 g of the inoculum (5,000 inoculant potential units) at a depth of 4 cm (Ren et al., 2015).

Approximately 6 weeks later, the watermelon seedlings and soil medium were transferred to a self-made continuous root exudate collection system for recovery cultivation 3 days (**Supplementary Figure S2**). The circulating pump was started, and distilled water dripped from above the plants into the pots. The flow rate was controlled at 1–1.5 L·h^-1^, and the soil medium was rinsed with enough water for 10 days. The column was removed after 10 days of the continuous collection of distilled water; the column was then eluted with 500 ml of deionized water and 200 ml of *n*-hexane at the same flow rate. Three eluents (biological replicates) were mixed. The eluent was then decompressed and steamed at 40 °C in a rotary evaporator until *n*-hexane was completely volatilized to obtain the dry matter of root exudates. Finally, the dry matter of the root exudates was dissolved in 10 ml of methanol for subsequent experiments.

### Experimental design and methods

The watermelon seeds were first washed and soaked in a water bath at constant temperature (55°C) for 30 min; the seeds were then incubated in an incubator at 28–30 °C until germination and then placed in 0.5 kg of watermelon soil that had been continuously cropped for 10 years (obtained from Liujiazhuang, Jimo (22°50′N, 108°17′E), Qingdao, China). The experiment was conducted under greenhouse conditions at Qingdao Agricultural University, and the greenhouse conditions were maintained at 25/15°C day/night, 14/10 h light/dark, and relative humidity of 70% (both day and night). Plants were watered at regular intervals or as needed.

After 6 weeks of cultivation of watermelon seedlings, the three types of root exudates collected were diluted 100 times (10 ml/L) and 200 times (5 ml/L), and each pot of watermelon seedlings was watered with 200 ml. The experiment included the following seven treatments: (1) M10 (10 ml/L); (2) M5 (5 ml/L); (3) V10 (10 ml/L); (4) V5 (5 ml/L); (5) C10 (10 ml/L); (6) C5 (5 ml/L); and (7) W (equivalent distilled water). The pots were arranged in a completely randomized block design with five replicates per treatment.

### Growth parameters

The length of the vine and the diameter of the stem of the watermelon were measured using a meter stick, and a chemical balance was used to determine the fresh weight of the aboveground and underground parts.

### Defense enzyme activities and malondialdehyde content

After the root exudates were treated for 21 d, 0.5 g of fresh leaves from different plants were weighed into a mortar, and 0.05 mol/L phosphoric acid buffer (pH 7.8) was added; the mixture was then ground in an ice bath and homogenized at −4 °C. After centrifugation at 4,000 rpm for 20 min, the supernatant was used to determine the content of superoxide dismutase (SOD), peroxidase (POD), catalase (CAT), and MDA. SOD was determined by the NBT method (Giannopolitis and Ries, 1977), POD was determined using the guaiacol method (Chance and Maehly, 1955), CAT was determined using the ultraviolet absorption method (Knörzer et al., 1996), and the MDA content was determined using the TBA method (Velikova et al., 2000).

### Soil enzyme activities

The activities of urease, sucrose, and CAT in the rhizosphere soil were measured on days 7, 14, and 21 after treatment. The soil urease activity was determined using phenol-sodium hypochlorite colorimetry, soil sucrase activity was determined using 3,5-dinitrosalicylic acid colorimetry, and soil CAT activity was determined using potassium permanganate titration (Guan, 1986).

### Microbial counts in rhizosphere soil

The plate dilution counting method was used to determine the number of microbes in the rhizosphere soil of the watermelon on day 21 after treatment. Beef extract peptone, Gause’s No. 1, and potato dextrose agar medium were used to isolate bacteria, actinomycetes, and fungi, respectively. The dilute plate counting method was used to measure the numbers of microbes (Zhao and He, 2002).

### Disease index

After the watermelon seedlings were treated with the root exudate extract for 21 days (After seed germination, watermelon seedlings cultivated for 6 weeks were treated with root secretion extract for 3 weeks, which corresponds to 9 weeks of growth), the disease severity of watermelon plants was scored independently on a scale of 0 to 4, with 0 indicating normal growth; 1 indicating mild wilting of leaves or stems (with the withered area ≤ 1/4 of the plant); 2 indicating the withered area accounting for 1/4–1/2 of the entire plant, and the presence of amber colloidal substances on the stem; and 3 indicating the withered area accounting for 1/2–3/4 of the entire plant, the presence of amber colloidal substances on the stem; and 4 indicating that the entire plant has withered or died (Ren et al., 2015).

The DI was calculated using a formula described in a previous study (Wang et al., 2020): DI (%) = [∑(Ni × Ri)/(Nt× 4)] × 100% (where Ri is the disease severity scale (Ri = 0, 1, 2, 3, 4), Ni is the number of diseased leaves of each disease severity scale, and Nt is the total number of leaves). The prevention and control effect (PCE) was calculated using the following formula: PCE (%) = [(DI of–control − DI of treatment)/DI of control] × 100%.

### Soil DNA extraction and high-throughput sequencing

After the watermelon seedlings were treated with the root exudate extract for 21 days, approximately 2 cm of soil on the surface of the pot was scraped away, and 2–15 cm of watermelon rhizosphere soil was collected. After mixing five duplicate samples, the roots, stones, and other debris were removed, and approximately 150 g of soil sample was immediately wrapped in a sterilized cloth bag with tin foil, frozen rapidly in liquid nitrogen, and stored at −80 °C for later use.

The Soil Genome DNA Extraction Kit (Tiangen, Beijing, China) was used to extract total rhizosphere soil DNA following the manufacturer’s instructions. The V3–V4 hypervariable region of the bacterial 16S rRNA gene was amplified using the primers 341F (5′-CCTAYGGGRBGCASCAG-3′) and 806R (5′-GGACTACNNGGGTATCTAAT-3′) (Thiergart et al., 2020). The resulting PCR products were subjected to 2% agarose gel electrophoresis and further purified using a gel recovery kit (Qiagen, Düsseldorf, Germany) according to the manufacturer’s protocol. The cDNA library was constructed using the TruSeq® DNA PCR-Free Sample Preparation Kit (Illumina, San Diego, USA). The library was quantified using a Qubit Fluorometer and qRT-PCR (Shanghai Genetic Biotech, China). The library was then subjected to high-throughput sequencing with an Illumina HiSeq 2500 platform (Illumina, San Diego, CA, USA) (Yang et al., 2022). The 16S rRNA gene sequences obtained in this study were deposited into the NCBI Sequence Read Archive database (Accession number: SRR24008190–SRR24008210).

Quality control of the molecular data was performed using the following steps. According to the barcode sequence and the PCR-amplified primer sequences, all paired-end raw reads were pre-processed using FLASH (Magoč and Salzberg, 2011). The raw data were quality filtered using Trimmomatic V 0.33 (http://www.usadellab.org/cms/index.php?page=trimmomatic), and then the barcodes and primer sequences were removed using Cutadapt v1.9.1 (Martin, 2011). Flash V1.2.11 was used to overlap and paste the high-quality reads of each sample to obtain clean reads (Edgar et al., 2011). Finally, UCHIME V8.1 was used to identify and remove the chimeric sequences to obtain the final valid reads (Edgar, 2013).

The operational taxonomic units (OTUs) with a 97% sequence similarity were clustered by UPARSE (version 7.1, http://drive5.com/uparse/) (Edgar, 2013). For high-throughput sequencing data, the Chao1, abundance-based coverage estimator (ACE), Shannon diversity index, and Simpson diversity index were calculated using QIIME software (version 1.7.0). QIIME software (version 1.7.0) was used to calculate the Unifrac distance and construct the sample clustering tree (Caporaso et al., 2010), and the rarefaction curve was drawn in R software (version 2.15.3). For beta diversity analysis, principal component analysis (PCA) plots were made using the ggplot2 package in R software to characterize variation in community composition among samples (Villanueva and Chen, 2019). Representative OTU sequences were analyzed using the Ribosomal Database Project (RDP) Classifier algorithm and GreenGene database for species annotation (the confidence threshold was set from 0.8 to 1). The taxonomic information was obtained, and the bacterial community composition (phylum, class, order, family, genus, and species levels) was determined.

### Data processing and statistical analysis

Microsoft Excel 2016 software (Microsoft Inc., Redmond, WA, USA) was used for the data processing, and the test data were analyzed using SPSS 18.0 software (SPSS, Inc., Chicago, USA). Statistically significant differences among treatments were determined by one-way ANOVA and least significant difference (LSD) calculations at the 95% confidence level.

## Results and analysis

### Effects of root exudates on the growth of watermelon

After 21 days of treatment, mycorrhizal watermelon root exudates could promote the growth of watermelon seedlings, and non-mycorrhizal watermelon root exudates inhibited the growth of watermelon seedlings (**Table 1**).

**Table 1:** Effects of root exudates on the growth of watermelon.

Treatments	Seeding length(cm)	Stem diameter(mm)	Fresh weight(g)	Dry weight(g)
M10	20.30 ± 0.73ab	5.03 ± 0.064bc	32.07 ± 0.33c	0.93 ± 0.0063ab
M5	22.53 ± 0.61a	5.90 ± 0.074a	41.43 ± 0.56a	1.01 ± 0.0079a
V10	22.03 ± 0.54ab	5.53 ± 0.071ab	37.23 ± 0.45ab	0.96 ± 0.015a
V5	20.83 ± 0.54ab	5.07 ± 0.022bc	34.80 ± 1.57bc	0.92 ± 0.0097ab
C10	14.73 ± 0.23c	4.00 ± 0.068d	22.97 ± 0.88de	0.83 ± 0.0056cd
C5	14.73 ± 0.32c	4.13 ± 0.075d	21.30 ± 0.97e	0.76 ± 0.015d
W	18.63 ± 0.44b	4.70 ± 0.094c	27.17 ± 1.21d	0.87 ± 0.0058bc

All data in the tables were expressed as means ± standard error. Different lowercase letters showing significant difference at p=0.05 using LSD test. M10 (10 ml/L) (root exudates of watermelon inoculated with *F. mosseae*); M5 (5 ml/L) (the same as M10); V10 (10 ml/L) (root exudates of watermelon inoculated with *G. versiforme*); V5 (5 ml/L) (the same as V10); C10 (10 ml/L) (root exudates of watermelon inoculated with sterilized inoculant); C5 (5 ml/L) (the same as C10); W (equivalent distilled water).

The growth of watermelon seedlings was promoted in the mycorrhizal watermelon root exudate treatment compared with the W treatment. The effect of M5 on the growth of watermelon seedlings was the most significant, and the length of the vine, stem diameter, fresh weight, and dry weight of watermelon plants were 20.9%, 25.5%, 54.5% and 15.9% higher in the M5 treatment than in the W treatment, respectively. The V10 treatment had the second strongest effect on the growth of watermelon seedlings, and the cranberry length, stem diameter, fresh weight, and dry weight were 18.3%, 17.7%, 37.0%, and 10.8% higher in the V10 treatment than in the W treatment, respectively. The application of non-mycorrhizal exudates inhibited the growth of watermelon seedlings, and the inhibitory effect of C5 was the most significant. The length of the vine, stem diameter, fresh weight, and dry weight were 20.9%, 12.1%, 21.6%, and 12.6% lower in the C5 treatment than in the W treatment, respectively (**Table 1**).

### Effects of root exudates on watermelon rhizosphere soil enzyme activities

The activities of sucrase, CAT, and urease in the rhizosphere soil of plants watered with the mycorrhizal root exudates were higher than those in the rhizosphere soil of plants in the W treatment with non-mycorrhizal root exudates, indicating that the mycorrhizal root exudates improved soil enzyme activities. After treatment with the root exudates for 7, 14, and 21 days, the activities of the enzymes in the M10, M5, V10, and V5 treatments were higher than those in the W treatment (**Table 2**). The activities of the three soil enzymes in the M5 treatment were significantly higher than those in the W treatment. After 7 days of treatment, soil sucrase, CAT, and urease activities were 83.0%, 11.3%, and 48.0% higher than those of W, respectively. After 14 days of treatment, the activities of the three enzymes were 55.7%, 10.6%, and 23.3% higher in the M5 treatment than in the W treatment, respectively. After 21 days of treatment, the activities of the three enzymes were 68.2%, 20.2%, and 16.1% higher in the M5 treatment than in the W treatment, respectively (**Table 2**).

**Table 2:** Effects of different root exudates on soil enzyme activities.

Treatments	Saccharase (mg/g)			Catalase (ml(0.1mol/L KMnO_4_)/(h·g))			Urease (mg/g)		
	7d	14d	21d	7d	14d	21d	7d	14d	21d
M10	11.2 ± 0.012c	11.59 ± 0.0097c	11.63 ± 0.0045c	2.94 ± 0.0075b	2.99 ± 0.0045b	3.12 ± 0.0048a	0.29 ± 0.0069c	0.33 ± 0.0048c	0.33 ± 0.0065c
M5	13.8 ± 0.023a	14.29 ± 0.012a	15.78 ± 0.0063a	3.09 ± 0.0063a	3.14 ± 0.0063a	3.16 ± 0.0096a	0.37 ± 0.0033a	0.37 ± 0.0053a	0.36 ± 0.0053a
V10	11.9 ± 0.014b	12.71 ± 0.011b	12.84 ± 0.013b	2.86 ± 0.011bc	2.97 ± 0.0047b	2.88 ± 0.0043c	0.33 ± 0.021b	0.34 ± 0.0044b	0.34 ± 0.0039b
V5	11.3 ± 0.011c	11.73 ± 0.0076c	12.39 ± 0.0057b	2.80 ± 0.0085c	2.99 ± 0.0075b	2.99 ± 0.016b	0.36 ± 0.0074a	0.33 ± 0.0047c	0.33 ± 0.011c
C10	7.0 ± 0.013e	8.20 ± 0.0065e	8.03 ± 0.0033e	2.66 ± 0.0048d	2.46 ± 0.013d	2.54 ± 0.0056d	0.21 ± 0.0054e	0.25 ± 0.0076e	0.23 ± 0.0096e
C5	6.0 ± 0.0034f	6.92 ± 0.0054f	6.55 ± 0.0047f	2.60 ± 0.015d	2.55 ± 0.014d	2.44 ± 0.0076e	0.18 ± 0.0065f	0.23 ± 0.011f	0.19 ± 0.0023f
W	9.1 ± 0.0089d	9.18 ± 0.012d	9.38 ± 0.013d	2.78 ± 0.0063c	2.84 ± 0.0075c	2.63 ± 0.0074d	0.25 ± 0.0078d	0.30 ± 0.0049d	0.31 ± 0.0042d

All data in the tables were expressed as means ± standard error. Different lowercase letters showing significant difference at p=0.05 using LSD test. M10 (10 ml/L) (root exudates of watermelon inoculated with *F. mosseae*); M5 (5 ml/L) (the same as M10); V10 (10 ml/L) (root exudates of watermelon inoculated with *G. versiforme*); V5 (5 ml/L) (the same as V10); C10 (10 ml/L) (root exudates of watermelon inoculated with sterilized inoculant); C5 (5 ml/L) (the same as C10); W (equivalent distilled water).

### Effects of root exudates on the number of microbes in the rhizosphere soil of watermelon plants

Root exudates can alter the structure of the soil microflora. After treatment with the root exudates from mycorrhizal watermelon seedlings, the number of actinomycetes and bacteria in the soil of the M10, M5, V10, and V5 groups increased significantly, and the number of fungi decreased significantly. The largest simultaneous increase in the number of bacteria and actinomycetes was observed in M5, and the number of bacteria and actinomycetes was 41.7% and 30.7% higher in the M5 treatment than in the W treatment, respectively. The most significant decrease in the number of actinomycetes was observed in the M10 treatment; specifically, the number of actinomycetes was 29.4% lower in the M10 treatment than in the W treatment. After treatment with the root exudates from non-mycorrhizal watermelon seedlings, the number of bacteria and actinomycetes in the rhizosphere soil of the plants decreased significantly, but the decrease was not significantly related to the concentration of root exudates applied; the number of fungi increased significantly, and greater root exudate concentrations were associated with more significant increases in the number of fungi. The number of fungi was 47.9% higher in the C5 treatment than in the W treatment (**Table 3**), In addition, the proportion of bacteria/actinomycetes and bacteria/fungi in the soil of the M10, M5, V10, and V5 groups was significantly higher than that in the W group; the bacteria/actinomycetes ratio in the V10 group was the highest, and the bacteria/fungi ratio in the M10 group was the highest (**Table 3**).

**Table 3:** Effects of different root exudates on amounts of soil microbial.

Treatments	Bacteria number (×10^5^CFU·g^–1^ DW)	Actinomycetes number (×10^3^CFU·g^–1^ DW)	Fungi number (×10^3^CFU·g^–1^ DW)	Bacteria/Actinomycetes	Bacteria/Fungi
M10	4.73 ± 0.33ab	3.57 ± 0.32a	3.83 ± 0.27e	1.33 ± 0.018b	1.2 ± 0.014a
M5	5.10 ± 0.40a	3.83 ± 0.26a	4.40 ± 0.32de	1.33 ± 0.010b	1.16 ± 0.0062b
V10	4.93 ± 0.34a	3.43 ± 0.29a	4.73 ± 0.27d	1.44 ± 0.039a	1.04 ± 0.0090c
V5	4.00 ± 0.26bc	3.57 ± 0.32a	4.80 ± 0.31d	1.12 ± 0.019cd	0.83 ± 0.016d
C10	2.57 ± 0.23d	2.40 ± 0.24c	6.53 ± 0.21b	1.07 ± 0.012d	0.39 ± 0.016f
C5	2.70 ± 0.38d	2.30 ± 0.14c	8.23 ± 0.25a	1.17 ± 0.075bc	0.33 ± 0.026g
W	3.60 ± 0.19c	2.93 ± 0.25b	5.57 ± 0.31c	1.23 ± 0.027bc	0.65 ± 0.0048e

All data in the tables were expressed as means ± standard error. Different lowercase letters showing significant difference at p=0.05 using LSD test. M10 (10 ml/L) (root exudates of watermelon inoculated with *F. mosseae*); M5 (5 ml/L) (the same as M10); V10 (10 ml/L) (root exudates of watermelon inoculated with *G. versiforme*); V5 (5 ml/L) (the same as V10); C10 (10 ml/L) (root exudates of watermelon inoculated with sterilized inoculant); C5 (5 ml/L) (the same as C10); W (equivalent distilled water).

### Effects of the root exudates on bacterial community structure and diversity in the rhizosphere soil

#### Data quality control

After sequencing, 267,670 bacterial gene sequences in each soil sample were obtained after removing the barcodes and primer sequences; 200,354 bacterial gene sequences were used for operational taxonomic unit (OTU) clustering and subsequent analyses after removing the target and chimera sequences. A total of 29,751, 27,770, 30,511, 29,330, 25,366, 28,599, and 29,028 available sequences were detected in the M10, M5, V10, V5, C10, and C5 treatments, respectively. The sequencing data of each sample are shown in **Supplementary Table 1**.

#### Sample complexity analysis

**Figure 1:**
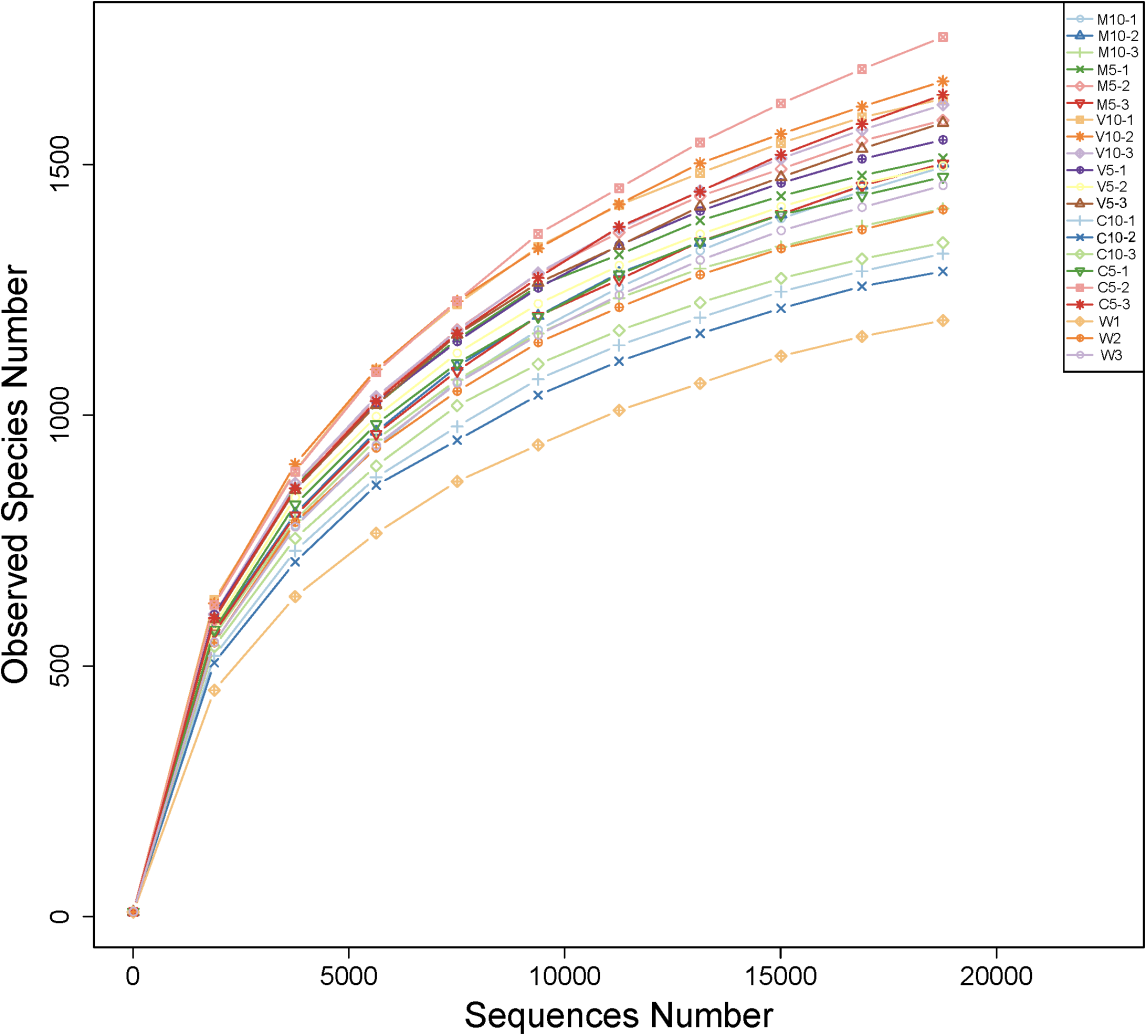
Rarefaction curves of OTUs in different treatments. M10 (10 ml/L) (root exudates of watermelon inoculated with *F. mosseae*); M5 (5 ml/L) (the same as M10); V10 (10 ml/L) (root exudates of watermelon inoculated with *G. versiforme*); V5 (5 ml/L) (the same as V10); C10 (10 ml/L) (root exudates of watermelon inoculated with sterilized inoculant); C5 (5 ml/L) (the same as C10); W (equivalent distilled water).

Bacterial abundance was determined using the alpha diversity estimates based on OTU abundance, including the Chao1 value, ACE value, Shannon index, and Simpson index. **Table 4** shows that the coverage rate of each treatment was more than 97%, indicating that the data provide an accurate representation of the species and structure of the community in the region. In addition, the Chao1 and ACE values of the C5 treatment were higher than those of the other treatments, indicating that low concentrations of non-mycorrhizal watermelon seedling root exudates are beneficial to increases in the diversity of rhizosphere soil microflora. No significant differences in the Simpson diversity index were observed among the six treatments (M10, M5, V10, V5, C10, and C5), indicating that the diversity was similar in the six treatments and the main OTUs of the community were similar. The Shannon diversity index was highest in the V10 treatment, indicating that soil microbes were most abundant when watermelon seedlings were inoculated with *G. versiforme* (**Table 4**). The rarefaction curve indicates whether the sequenced material adequately represents all populations and reflects species richness. The 21 rarefaction curves for the seven treatments (M10, M5, V10, V5, C10, C5, and W) are shown in **Figure 1**. The rarefaction curve for each sample gradually plateaued, but saturation was not achieved. The results showed that the sequencing data were robust, and more data would only generate a few new species (OTUs). The sample size of the database is sufficient for characterizing microbial diversity in the environment (**Figure 1**).

**Table 4:** Alpha diversities in different inoculation treatments.

Treatments	Shannon	Simpson	Chao1	ACE	Goods coverage
M10	8.46 ± 0.035bc	0.993 ± 0.00a	1837.38 ± 124.84abc	1836.44 ± 105.68abc	0.9780 ± 0.00ab
M5	8.59 ± 0.043ab	0.993 ± 0.00a	1796.49 ± 64.43abc	1835.28 ± 59.20abc	0.9797 ± 0.00ab
V10	8.78 ± 0.041a	0.994 ± 0.00a	1934.45 ± 70.85ab	1996.29 ± 57.58a	0.9773 ± 0.00ab
V5	8.66 ± 0.020ab	0.994 ± 0.00a	1814.57 ± 121.64abc	1859.18 ± 76.37ab	0.9793 ± 0.00ab
C10	8.21 ± 0.043cd	0.991 ± 0.00a	1498.19 ± 21.82c	1553.42 ± 15.22c	0.9837 ± 0.00a
C5	8.61 ± 0.068ab	0.993 ± 0.00a	2073.30 ± 203.34a	2097.41 ± 188.27a	0.9740 ± 0.00b
W	8.09 ± 0.25d	0.986 ± 0.00b	1586.70 ± 110.24bc	1659.08 ± 93.38bc	0.9813 ± 0.00a

All data in the tables were expressed as means ± standard error. Different lowercase letters showing significant difference at p=0.05 using LSD test. M10 (10 ml/L) (root exudates of watermelon inoculated with *F. mosseae*); M5 (5 ml/L) (the same as M10); V10 (10 ml/L) (root exudates of watermelon inoculated with *G. versiforme*); V5 (5 ml/L) (the same as V10); C10 (10 ml/L) (root exudates of watermelon inoculated with sterilized inoculant); C5 (5 ml/L) (the same as C10); W (equivalent distilled water).

### OTU analysis and species notes

**Figure 2:**
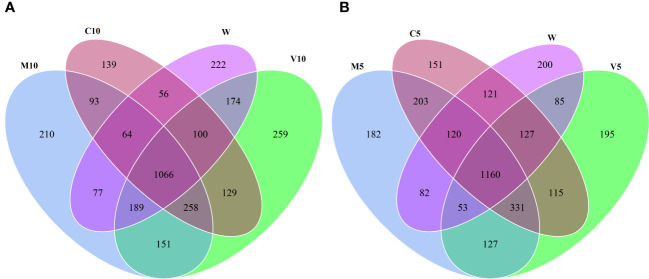
Venn plot of the number of OTUs under high concentration **(A)** and low concentration **(B)** root exudates treatments. M10 (10 ml/L) (root exudates of watermelon inoculated with *F. mosseae*); M5 (5 ml/L) (the same as M10); V10 (10 ml/L) (root exudates of watermelon inoculated with *G. versiforme*); V5 (5 ml/L) (the same as V10); C10 (10 ml/L) (root exudates of watermelon inoculated with sterilized inoculant); C5 (5 ml/L) (the same as C10); W (equivalent distilled water).

Based on the OTU clustering analysis, common and unique OTUs between different processes were analyzed, and Venn diagrams were made. The effects of root exudates applied at the same concentrations were compared, and the results are shown in **Figure 2**. Each circle in **Figure 2** represents a process. The number of circles and overlapping portions of the circles represent the number of OTUs shared between processes, and the number of overlapping portions represents the unique number of OTUs for each process. When the concentrations of the root exudates were low, there were 3,187 unique bacterial OTUs, of which 93 OTUs were shared between the C10 and M10 treatments; 129 OTUs were shared between the C10 and V10 treatments, 151 OTUs were shared between the V10 and M10 treatments, and 56 OTUs were shared between the C10 and W treatments (**Figure 2A**). When the root exudate concentrations were high, there were 3,252 unique bacterial OTUs, of which 203 OTUs were shared between the C5 and M5 treatments, 115 OTUs were shared between the C5 and V5 treatments, 127 OTUs were shared between the V5 and M5 treatments, and 121 OTUs were shared between the C5 and W treatments (**Figure 2B**). Treatments in which the same concentrations of root exudates were applied were compared with the W treatment using the Sorensen similarity index. The results showed that the similarity among all treatments was more than 65%, and the similarity of each treatment with the W treatment was between 0.67 and 0.72. **Table 5** shows that higher concentrations of root exudates in the mycorrhizal watermelon seedlings correspond to greater similarity between the treatment group and W treatment; higher concentrations of root exudates in the non-mycorrhizal watermelon seedlings result in less similarity of the W treatment with the other treatments. The similarity between V5 and C5 was 0.77, and that between M5 and C5 was 0.79 (**Table 5**).

**Table 5:** Comparison of the degree of similarity between each sample in the horizontal direction.

	M10	M5	V10	V5	C10	C5	W
M10	1						
M5		1					
V10	0.75		1				
V5		0.75		1			
C10	0.74		0.73		1		
C5		0.79		0.77		1	
W	0.69	0.67	0.72	0.69	0.67	0.72	1

Different lowercase letters showing significant difference at p=0.05 using LSD test. M10 (10 ml/L) (root exudates of watermelon inoculated with *F. mosseae*); M5 (5 ml/L) (the same as M10); V10 (10 ml/L) (root exudates of watermelon inoculated with *G. versiforme*); V5 (5 ml/L) (the same as V10); C10 (10 ml/L) (root exudates of watermelon inoculated with sterilized inoculant); C5 (5 ml/L) (the same as C10); W (equivalent distilled water).

### Species relative abundance analysis

**Figure 3:**
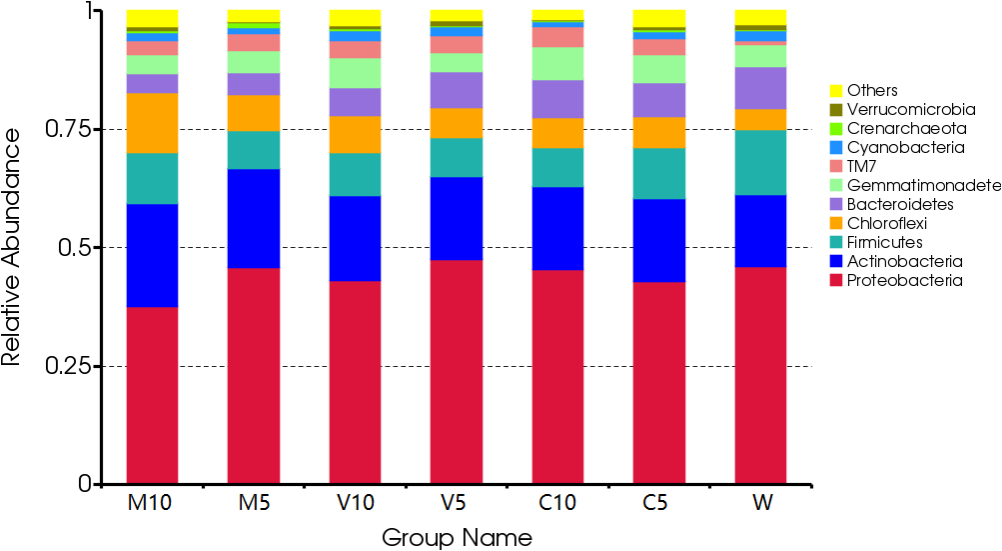
Effects of different treatments on the composition and structure of the soil bacterial community at the phylum level. M10 (10 ml/L) (root exudates of watermelon inoculated with *F. mosseae*); M5 (5 ml/L) (the same as M10); V10 (10 ml/L) (root exudates of watermelon inoculated with *G. versiforme*); V5 (5 ml/L) (the same as V10); C10 (10 ml/L) (root exudates of watermelon inoculated with sterilized inoculant); C5 (5 ml/L) (the same as C10); W (equivalent distilled water).

After irrigating with different concentrations of root exudates, the dominant bacteria in the rhizosphere soil did not change at the phylum level, and Proteobacteria were the dominant bacteria, accounting for more than 37.5% of all bacteria. The proportion of Proteobacteria and Bacteroides increased in the soil as the concentration of root exudates in the mycorrhizal watermelon seedlings decreased and the proportion of Actinomycetes, Firmicutes, and Chloroflexi decreased. Proteobacteria accounted for 46.07% of all bacteria in W. After irrigating with the V5 root exudates, their relative abundance increased to 47.54%; Bacteroides accounted for 8.8% of all bacteria in the W treatment, and the relative abundance of the M10 root exudates decreased to 4.01% after irrigation. Actinomycetes accounted for 15.17% of all bacteria in the W treatment, and their relative abundance increased to 21.91% after irrigating with the M10 root exudates. Firmicutes accounted for 13.67% of all bacteria in the W treatment, but their relative abundance decreased to 7.95% after irrigation with the M5 root exudates. Chloroflexi accounted for 4.53% of all bacteria in the W treatment, and their relative abundance increased to 12.84% after irrigating with the M10 root exudates. In addition, after irrigation with the C10 root exudates, the proportion of Gemmatimonadetes increased from 4.60% in the W treatment to 6.90% (**Figure 3**).

**Figure 4:**
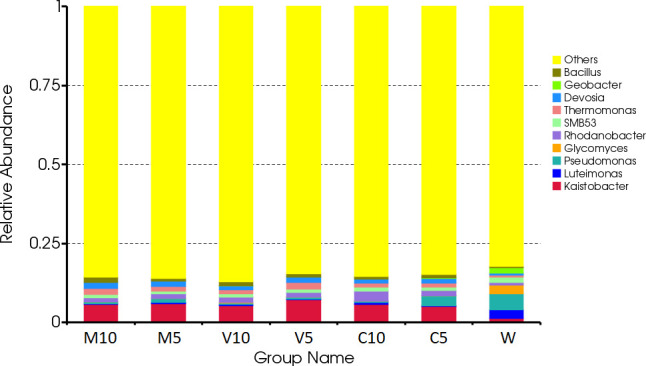
Effects of different treatments on the composition and structure of the soil bacterial community at the genus level. M10 (10 ml/L) (root exudates of watermelon inoculated with *F. mosseae*); M5 (5 ml/L) (the same as M10); V10 (10 ml/L) (root exudates of watermelon inoculated with *G. versiforme*); V5 (5 ml/L) (the same as V10); C10 (10 ml/L) (root exudates of watermelon inoculated with sterilized inoculant); C5 (5 ml/L) (the same as C10); W (equivalent distilled water).

**Figure 5:**
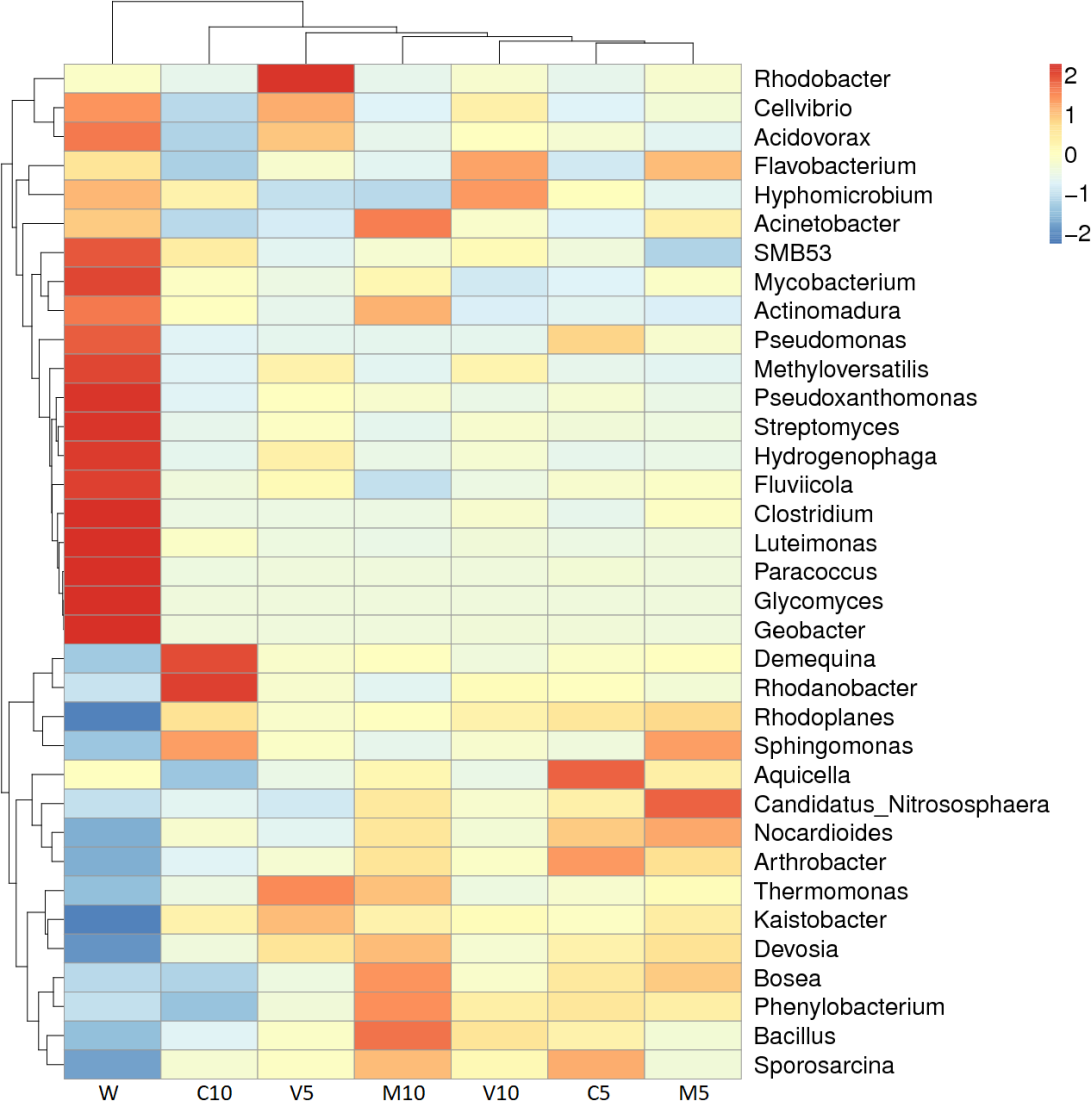
Species richness dendrogram. M10 (10 ml/L) (root exudates of watermelon inoculated with *F. mosseae*); M5 (5 ml/L) (the same as M10); V10 (10 ml/L) (root exudates of watermelon inoculated with *G. versiforme*); V5 (5 ml/L) (the same as V10); C10 (10 ml/L) (root exudates of watermelon inoculated with sterilized inoculant); C5 (5 ml/L) (the same as C10); W (equivalent distilled water). The horizontal axis shows the sample information, and the vertical axis shows the species information. The species clustering tree is shown on the left, the sample clustering tree is shown above, and the corresponding value of the middle heat map is the value obtained after the standardized processing of the relative abundance of species in each row.

The dominant bacteria in the W treatment at the genus level were *Luteimonas*, *Pseudomonas*, and *Glycomyces*. After watering the root exudates of the watermelon seedlings, the dominant bacteria were *Kaistobacter*, *Rhodanobacter*, *Thermomonas*, *Devosia*, and *Bacillus*. After irrigating with different concentrations of the mycorrhizal root exudates, the abundances of *Kaistobacter* in the M10, M5, V10, and V5 treatments were 5.76%, 5.98%, 5.29%, and 7.17%, respectively. The abundance of *Kaistobacter* was 4.31 times higher in V5 than in W, and the abundance of *Devosia* in M10, M5, V10, and V5 was 1.93%, 1.76%, 1.38%, and 1.73%, respectively. The abundance of *Kaistobacter* was 1.88 times higher in M10 than in W. In addition, the abundance of *Kaistobacter* was higher after irrigation of the root exudates of non-mycorrhizal watermelon seedlings compared with that in the W treatment, but the proportion of *Kaistobacter* was lower in C10 or C5 than in M10, M5, V10, and V5 (**Figures 4** and **5**).

### Multi-sample comparative analysis

**Figure 6:**
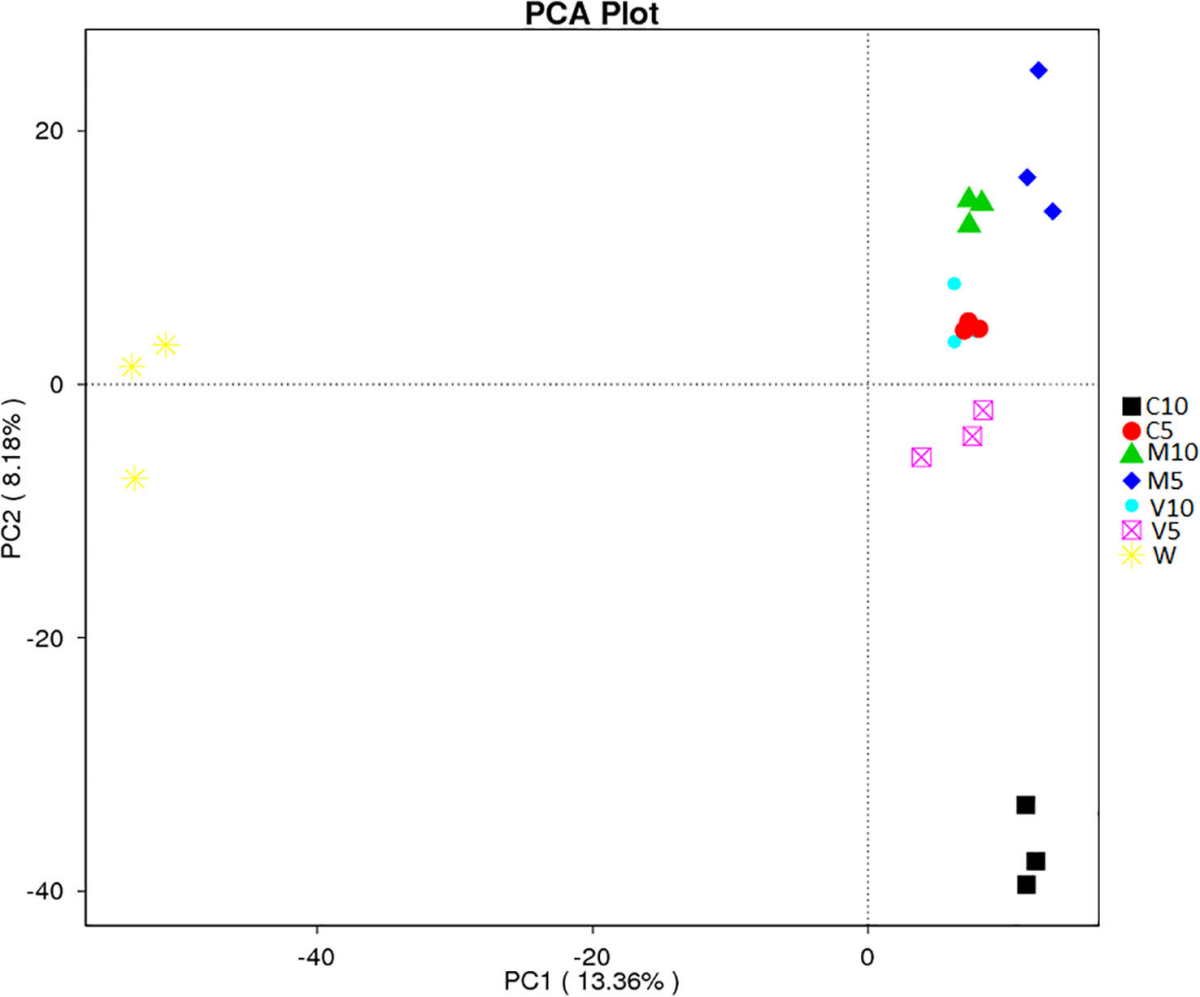
β-diversity analysis of the microbial community across each genus by PCA for the seven treatments (C10, C5, M10, M5, V10, V5, and W). M10 (10 ml/L) (root exudates of watermelon inoculated with *F. mosseae*); M5 (5 ml/L) (the same as M10); V10 (10 ml/L) (root exudates of watermelon inoculated with *G. versiforme*); V5 (5 ml/L) (the same as V10); C10 (10 ml/L) (root exudates of watermelon inoculated with sterilized inoculant); C5 (5 ml/L) (the same as C10); W (equivalent distilled water). The abscissa shows the first principal component, and the percentage indicates the contribution of the first principal component to the sample difference. The ordinate indicates the second principal component, and the percentage indicates the contribution of the second principal component to the sample difference. Each symbol in the figure indicates a sample, and samples from the same group are indicated by the same color.

PCA uses variance decomposition to reduce the dimensions of multidimensional data and extract the most important elements from the data. PCA extracts two coordinate axes that reflect the differences between samples to the greatest extent and reflects the differences in the multi-dimensional data on a two-dimensional coordinate map. The distance between samples in the PCA plot is positively related to the similarity in community composition. Separation was observed between the rhizosphere soil samples of different treatment groups; the contribution rate of the first principal component (PC1) was 13.36%, the contribution rate of the second principal component (PC2) was 8.18%, and the cumulative contribution rate of PC1 and PC2 was 21.54% (**Figure 6**). The principal components of each group were relatively clustered, but overlap was observed among the two V10 replicates and the three C5 replicates. In addition, the distance between C5, V10, V5, M10, and M5 was relatively low, and they were all located far from W, indicating that root exudates had a strong effect on soil bacterial community composition, whereas the water treatment had almost no effect. However, the distance between C10 and C5 was relatively large, indicating that soil bacterial community composition was more sensitive to the concentration of watermelon root exudates inoculated with *G. versiforme* (**Figure 6**).

**Figure 7:**
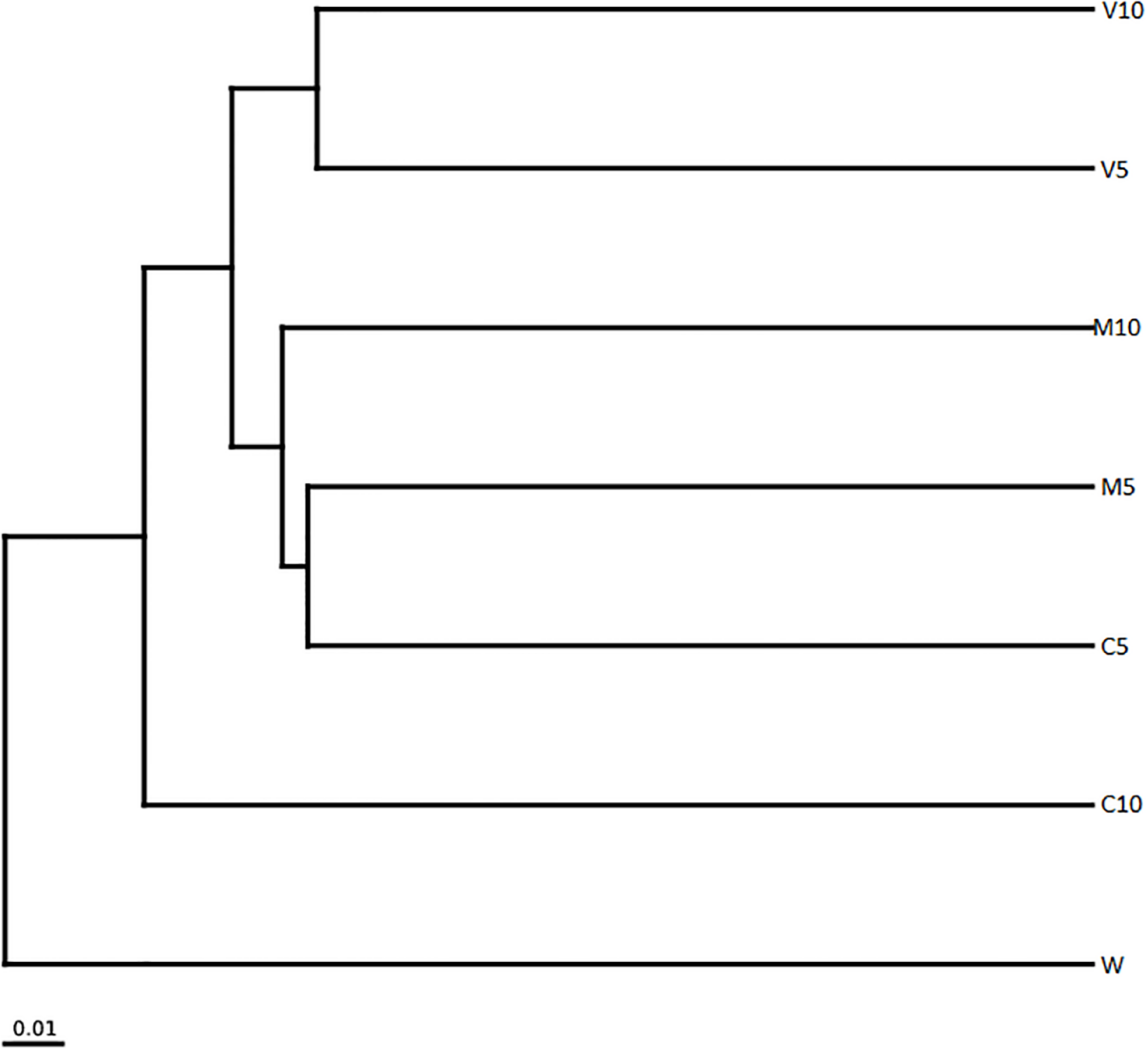
Cluster analysis of the dominant genera in different soil samples based on unweighted UniFrac distances. M10 (10 ml/L) (root exudates of watermelon inoculated with *F. mosseae*); M5 (5 ml/L) (the same as M10); V10 (10 ml/L) (root exudates of watermelon inoculated with *G. versiforme*); V5 (5 ml/L) (the same as V10); C10 (10 ml/L) (root exudates of watermelon inoculated with sterilized inoculant); C5 (5 ml/L) (the same as C10); W (equivalent distilled water).

The clustering tree for the sample clustering analysis showed that all samples were clustered into four groups (**Figure 7**). W independently formed branches on the clustering tree. C10 also formed branches on the clustering tree; V10 and V5 were clustered together, and M10, M5, and C5 were clustered together. W was the most distant from the other samples, and the community composition of W differed from that of the other treatment groups. These results show that irrigation with root exudates altered the composition of the watermelon rhizosphere soil microbial community, and the most significant change was observed in W. The effects of root exudates on soil microbial community composition in the treated watermelon seedlings were similar (V10 and V5, M10 and M5), and the similarity of the non-mycorrhizal watermelon seedling root exudates with the soil microbial community composition was relatively weak.

### Effects of root exudates on watermelon Fusarium wilt

All the plants irrigated with a high concentration (C5) of non-mycorrhizal watermelon seedling root exudates were infected. The infection rate of the plants was higher in the C10 group than in W, and the infection rate of the plants in the M10, M5, V10, and V5 treatment groups was lower than that in W. Watering with the root exudates from the mycorrhizal watermelon seedlings reduced the DI of Fusarium wilt by 6.7–30% compared with W, and the control effect was 11.2–50%. The control effect was strongest in M5, followed by V10, V5, and M10; the PCE of M5 was 50%, and that of V10 was 33% (**Table 6**).

**Table 6:** Effects of different root exudates on *Fusarium* disease status.

Treatments	Incidence rate (%)	Disease index (DI)	PCE (%)
M10	60	53.3	11.2
M5	40	30	50
V10	40	40	33.3
V5	50	50	16.7
C10	80	60	0
C5	100	83.3	-38.8
W	60	60	0

Different lowercase letters showing significant difference at p=0.05 using LSD test. M10 (10 ml/L) (root exudates of watermelon inoculated with *F. mosseae*); M5 (5 ml/L) (the same as M10); V10 (10 ml/L) (root exudates of watermelon inoculated with *G. versiforme*); V5 (5 ml/L) (the same as V10); C10 (10 ml/L) (root exudates of watermelon inoculated with sterilized inoculant); C5 (5 ml/L) (the same as C10); W (equivalent distilled water).

## Discussion

The health of plants depends on the stability and balance of the plant–soil–microbial system, and microorganisms play key roles in this system (Hartmann et al., 2008), as they degrade macromolecular substances in the soil into smaller molecules and convert insoluble nutrients into forms available to plants. Some beneficial microorganisms also antagonize soil-borne pathogens and improve plant resistance (Wang et al., 2017). In addition, plant root exudates promote or inhibit the growth of pathogenic bacteria during interactions between watermelon plants and soil pathogenic bacteria (Dixon, 2001; D’Auria and Gershenzon, 2005). Improving the rhizosphere soil environment via inoculation with beneficial microorganisms has become an important approach for overcoming the challenges of the continuous cropping of watermelon (Xue et al., 2019). AMF are widely used because they have direct and indirect beneficial effects on plant growth and development, environmental stress tolerance, and sustainable agricultural development (Gutjahr and Parniske, 2013; Jiang et al., 2017; Choi et al., 2020). Fusarium wilt is caused by a soil-borne fungal pathogen that survives in soil and crop debris for many years and enters plants through root wounds. Previous studies have shown that colonization by AMF alleviates tomato *F. oxysporum* blight (Nahiyan and Matsubara, 2012; Hashem et al., 2021). In addition, plant root exudates are the first signaling substances sensed by AMF, and this promotes the growth of AMF spores (Yin et al., 2016). AMF also release substances into the rhizosphere, which affects the composition of the host–plant root exudates (Lü and Wu, 2018).

### Effects of root exudates from mycorrhizal watermelon seedlings on plants (growth and antioxidant enzymes)

AMF are widely recognized for their ability to promote plant growth and increase yield; they are useful biological control agents (Eke et al., 2016; Spagnoletti et al., 2017; Spagnoletti et al., 2018). AMF promote plant growth mainly because they alter the composition of root exudates; produce phenols, antibiotics, and other inhibitory compounds; and induce the plant defense system (Majewska et al., 2017; Spagnoletti et al., 2017). In this study, the mycorrhizal watermelon root exudates promoted the growth of watermelon seedlings; non-mycorrhizal watermelon root exudates inhibited the growth of watermelon seedlings, and M5 and V10 had the most significant effect. Additionally, vine length, stem diameter, fresh weight, and dry weight were significantly higher in M5 and V10 than in W (**Table 1**). AMF provide host plants with nutrients, such as inorganic phosphate, and recruit beneficial rhizosphere bacteria and fungi that promote nitrogen and phosphorus uptake by plants (Jiang et al., 2017; Luginbuehl et al., 2017), inhibit soil-borne pathogens, and prevent soil microbial imbalances and nutrient deficiencies during continuous cropping (Liu et al., 2020).

The antioxidant enzyme system is an important reactive oxygen scavenging system in plants that plays an important role in environmental stress (Suneja et al., 2017). The activities of CAT, SOD, POD, and other antioxidant enzymes are closely related to disease resistance. MDA is an indicator of the degree of membrane lipid peroxidation, and the MDA level reflects the degree of injury caused by stress (Liu and Huang, 2000). In this study, the activities of the antioxidant enzymes were higher in M10, M5, V10, and V5 than in C10, C5, and W, and the change in the MDA content was opposite to the change in the activities of defensive enzymes (**Supplementary Figure S1**). These results indicate that the root exudates of mycorrhizal plants improved the utilization efficiency of soil nutrients, enhanced the reactive oxygen species scavenging ability, and enhanced the resistance of plants to stress.

### Effects of root exudates from mycorrhizal watermelon seedlings on soil enzyme activities

The role of soil enzymes is to balance carbon and nitrogen in the soil and provide nutrients for plant growth. Soil enzyme activity reflects the productivity of the soil and the health of the soil ecosystem (Zhou et al., 2013). Among the soil enzymes, the activity of urease reflects the nitrogen supply capacity of the soil (Dumas-Gaudot et al., 1992), and CAT is the main redox enzyme in soil, which reflects the intensity of the soil oxidation processes (Doornbos et al., 2012). In this study, the activities of watermelon sucrase, CAT, and urease were higher in the root exudates from M10, M5, V10, and V5 than in the root exudates from W, and the activities of these three enzymes were lower in the C10 and C5 treatment groups than in the W group, indicating that the soil enzyme activities in watermelon greenhouse soil decreased after 10 years of continuous cropping. Zhao et al. (2010) reported that the long-term continuous cropping of watermelon has an inhibitory effect on soil proteases and polyphenol oxidases, and the activities of urease and glucoamylase significantly increased after inoculation of *G.* versiforme. In this study, the root exudates of mycorrhizal watermelon also increased the enzyme activities in the rhizosphere soil after inoculating *F. mosseae* and *G.* versiforme, which was consistent with the results of Zhao et al. (2010). They reported that 2–4 mm and 1–2 mm of soil water-stable aggregates increased in continuously cropped soils inoculated with AMF, which resulted in the greater stability of the aggregates in the inoculated soil than in the treatment group (Lǚ et al., 2019). The increases in soil enzyme activities contribute to increases in the soil carbon, nitrogen, and phosphorus content, as well as improvements in soil fertility (Lian et al., 2019). In addition, increases in soil enzyme activities accelerate the decomposition of organic matter, provide nutrients needed for microbial growth, increase the growth rate of microorganisms, and improve the growth environment of microorganisms. At the same time, microorganisms continuously secrete enzymes into the soil, and this promotes further increases in soil enzyme activities (Zhou et al., 2020). However, some studies have reported that the root exudates of strawberries planted in non-continuously cropped soil inhibit the activities of soil CAT and urease regardless of whether the soil was inoculated with *F. mosseae* (Hu et al., 2016). The results of this study showed that whether AMF has a positive effect on soil enzyme activities in continuously cropped soil depends on the plant and AMF species.

### Effects of the root exudates of mycorrhizal watermelon inhibit disease caused by *Fusarium oxysporum*

Watermelon is highly sensitive to infection by FON, which results in Fusarium wilt. The results of this study show that the root exudates of the mycorrhizal watermelon seedlings reduced the incidence and DI of watermelon Fusarium wilt, and the control effect was 11.2–50%. In contrast, watermelon root exudates in continuously cropped soil had no control effect, which was consistent with the results of previous studies of asparagus, tomato (*Solanum lycopersicum*), and other plants (Nahiyan and Matsubara, 2012; Hernández-Montiel et al., 2013). Some specific components in the root exudates of mycorrhizal plants are thought to play a key role in the control of soil-borne diseases, and colonization by AMF promotes the secretion of coumaric acid and malic acid in the root exudates of *F. oxysporum* (Ren et al., 2015). In addition, phenolic acid, which accumulates in the root exudates of mycorrhizal plants, inhibits spore germination by *F. oxysporum* (Ren et al., 2008; Steinkellner et al., 2008). Wu et al. (2008) reported that *p*-hydroxybenzoic acid, phthalic acid, gallic acid, coumarinic acid, cinnamic acid, ferulic acid, salicylic acid, and cinnamic acid in the root exudates have antifungal effects and effectively destroy plant pathogens. Therefore, AMF colonization controls the occurrence of watermelon Fusarium wilt by altering the composition of root exudates; however, Al-Hmoud and Al-Momany (2015) reported no significant difference between tomato plants inoculated with *F. oxysporum* and mycorrhizal control plants, demonstrating that the ideal control effect can be achieved via the inoculation of the appropriate AMF for each crop.

### Effects of the root exudates of mycorrhizal watermelon on soil microorganisms (quantity and species)

Soil-borne pathogens, including fungi, bacteria, nematodes, and viruses, can have major deleterious effects on plants in soils that are continuously cropped. One of the main changes observed in continuously cropped soil is the transformation of the structure of the soil microbial community from a “bacterial type” to a “fungal type” (Wu et al., 2009). A higher fungi/bacteria ratio in continuously cropped soil is associated with lower soil fertility. Root exudates indirectly affect the types of rhizosphere microbes by altering the rhizosphere environment, and they play an important role in shaping the soil microbial community (Doornbos et al., 2012). In this study, the total number of microorganisms, bacteria/actinomycetes, and the bacteria/fungi ratio were higher in the M10, M5, V10, and V5 treatment groups than in the C10 and C5 groups, indicating that AMF inoculation increased the bacteria/fungi ratio in the soil by promoting the growth of beneficial bacteria and inhibiting pathogenic fungi, which is consistent with the results of a previous study in which watermelon was inoculated with *Saccharomyces cerevisiae* in continuously cropped soil (Zhao et al., 2010). Similarly, the number of soil bacteria and total microorganisms in mycorrhizal peach seedlings is high under continuously cropped conditions, and the number of soil fungi is low (Lǚ et al., 2019) because the continuous cropping of watermelon alters the soil microbial flora. The length of the continuous cropping period is inversely related to the number of bacteria and beneficial actinomycetes in the soil but positively relative to the number of fungi (Zhao et al., 2008). However, AMF may become the dominant strain in the soil when the roots of watermelon seedlings are inoculated with AMF, which inhibits the growth of other fungi and results in a decrease in the number of fungi in the soil, an increase in the bacteria/fungi ratio, and an increase in soil fertility. The number of soil actinomycetes was higher in the M10, M5, V10, and V5 treatment groups than in the C10 and C5 groups. However, Sun et al. (2017) reported that the number of soil actinomycetes was lower in cucumber when it was inoculated with *F. mosseae* than in the control (cucumbers that had not been inoculated in non-continuously cropped soil). This might stem from differences in the AMF variety, host plant, or soil type. Similar results were obtained by Garbeva et al. (2004).

### Mycorrhizal watermelon root exudates affect the diversity of the soil bacterial community

Plant root exudates alter the diversity of microorganisms in the rhizosphere soil; thus, changes in the quantity and type of root exudates indirectly alter the composition of the soil microbial community. Previous studies have shown that inoculation with AMF alters the composition of the rhizosphere microbial community (Ren et al., 2008; Steinkellner et al., 2008). In our study, no changes in the dominant bacterial phyla in the rhizosphere soil were observed after watermelon seedlings were watered with root exudates; Proteobacteria, Actinomyces, and Firmicutes were the dominant groups both before and after the application of root exudates. These findings are consistent with the results of previous studies. The type of root exudate might affect changes in soil bacterial composition and diversity. Organic acids and amino acids generally reduce microbial community diversity, and a mixture of amino acids and nitrogen-containing compounds increases the severity of disease symptoms, which, in turn, leads to a decrease in bacterial community diversity (Gu et al., 2020). In addition, the physiological state of the plant can affect soil microbial community composition, including the jasmonic acid signaling pathways involved in the responses to certain bacteria that affect the composition of the rhizosphere bacterial community (Carvalhais et al., 2013).

The results of this study show that the enriched microorganisms in the rhizosphere soil of the plants decreased sharply, and the bacteria multiplied in large numbers after irrigation with the root exudates. C10 had the lowest diversity among the bacterial populations, and *Demequina* and *Rhodanobacter* were the most abundant bacteria; this might stem from the high concentration of root exudates, which had a toxic effect on the rhizosphere soil.

In the environment, the soil microecosystem comprising the plant, soil, and rhizosphere microorganisms is extremely complex. This experiment showed that mycorrhizal watermelon root exudates increased the diversity of the rhizosphere soil bacterial community, decreased the abundance of plant pathogenic bacteria, and improved the structure of the rhizosphere soil bacterial community, which might be related to changes in the root exudates after inoculation with AMF. In a subsequent study, gas chromatography–mass spectrometry will be used to analyze the main components of the mycorrhizal and non-mycorrhizal root exudates to identify specific substances.

## Conclusion

The mycorrhizal watermelon root exudates could alter the composition of bacteria in watermelon rhizosphere soil, improve soil enzyme activity, and promote the transformation of watermelon rhizosphere soil from “fungal-type” soil to “bacterial-type” soil, which increases the resistance of watermelon to Fusarium wilt. Therefore, the root exudates of mycorrhizal plants can be used to control soil-borne diseases. The results of this study provide insights that will aid future studies of the role of AMF in agricultural production and the prevention of soil-borne disease. Additional research is needed to characterize the inhibitory effects of different chemical components of mycorrhizal watermelon root exudates on Fusarium wilt.

## Data availability statement

The datasets presented in this study can be found in online repositories. The names of the repository/repositories and accession number(s) can be found below: https://www.ncbi.nlm.nih.gov/genbank/, SRR24008190–SRR24008210.

## Author contributions

ML designed the experiment. X-YH and C-SZ performed the experiment. WL participated in writing the manuscript. WL and S-XG participated in revising the manuscript. All authors contributed to the article and approved the submitted version.
