# Supplementary material for: Control effect of root exudates from mycorrhizal watermelon seedlings on Fusarium wilt and the bacterial community in continuously cropped soil
Source: Front Plant Sci. 2023 Sep 11;14:1225897. doi: 10.3389/fpls.2023.1225897 (PMC10520283; doi:10.3389/fpls.2023.1225897)
Supplement: Supplementary file 1 [file DataSheet_1.docx]

***Supplementary material***

**Control effect of root exudates from mycorrhizal watermelon seedlings on Fusarium wilt and the bacterial community in continuously cropped soil**

Wei Li^a,b^, Xue-Yi Hu^a,b^, Cheng-Shang Zhu^a,b^, Shao-xia Guo^a,b^ ,Min Li^b,*^

^a^College of Landscape Architecture and Forestry, Qingdao Agricultural University, Qingdao, Shandong. P. R. China.

^b^Institute of Mycorrhizal Biotechnology, Qingdao Agricultural University, Qingdao, Shandong. P. R. China.

* Corresponding author. E-mail: minli@qau.edu.cn

ORCID: LI Min (0000-0003-1466-6324)

**Supplementary Table 1 Sample data processed**

|  | Quality control | Average length | Nontarget sequence | Chimera | Available sequence |
| --- | --- | --- | --- | --- | --- |
| M10 | 38877 | 413 | 8234 | 891 | 29751 |
| M5 | 36895 | 413 | 7967 | 1157 | 27770 |
| V10 | 41014 | 414 | 9299 | 1205 | 30511 |
| V5 | 39151 | 415 | 8839 | 983 | 29330 |
| C10 | 33998 | 415 | 7897 | 736 | 25366 |
| C5 | 38131 | 414 | 8475 | 1057 | 28599 |
| W | 39604 | 417 | 9378 | 1198 | 29028 |

Note: All data in the tables were expressed as means ± standard error. Different lowercase letters showing significant difference at p=0.05 using LSD test. M10 (10 mL/L) (root exudates of watermelon inoculated with *F. mosseae*); M5 (5 mL/L) (the same as M10); V10 (10 mL/L) (root exudates of watermelon inoculated with *G. versiforme*); V5 (5 mL/L) (the same as V10); C10 (10 mL/L) (root exudates of watermelon inoculated with sterilized inoculant); C5 (5 mL/L) (the same as C10); W (equivalent distilled water)


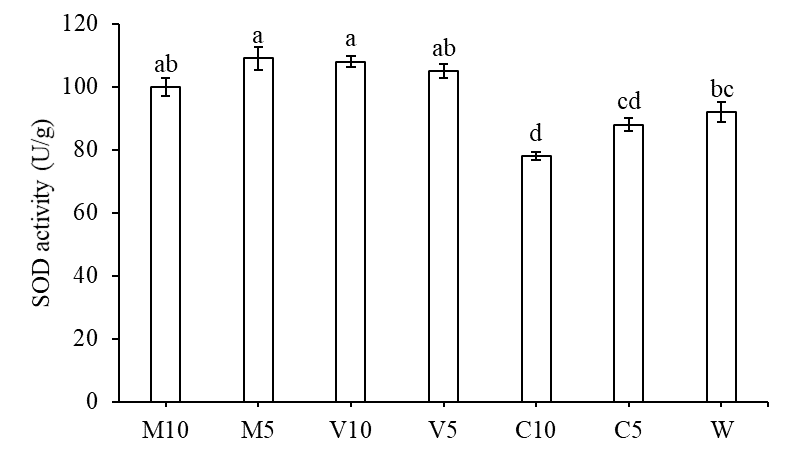

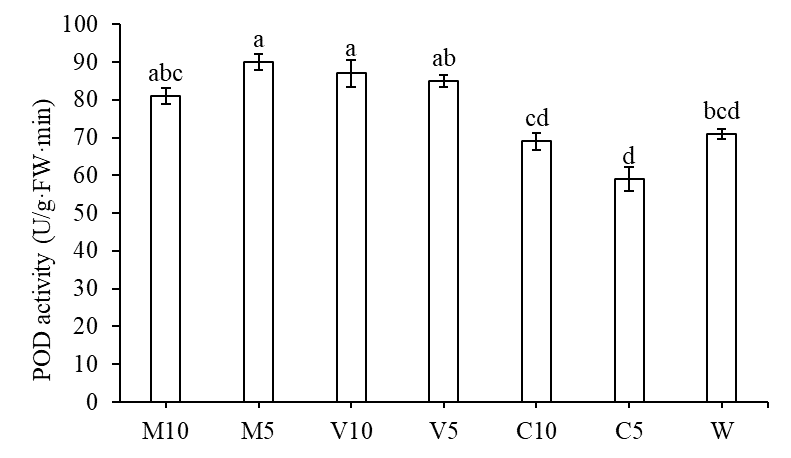


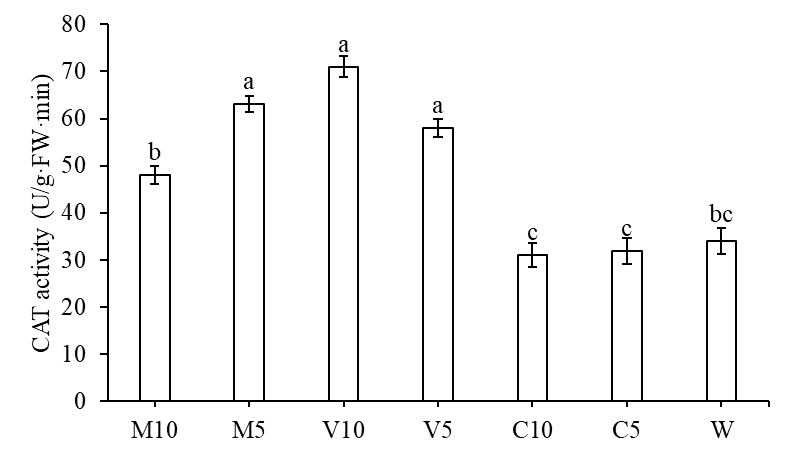

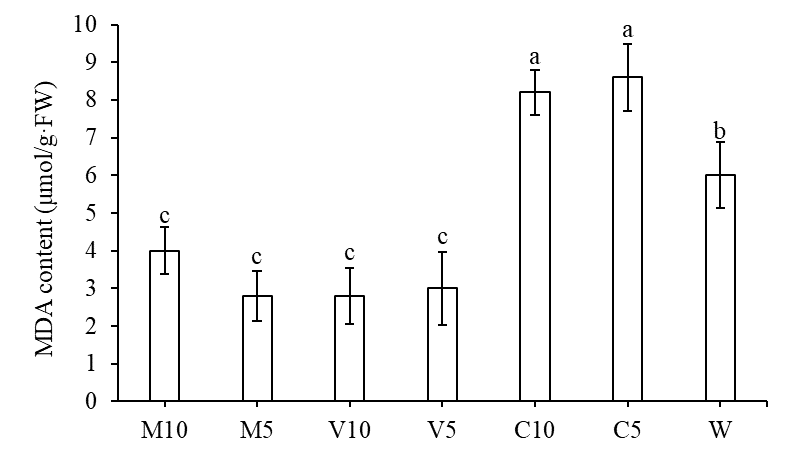


**Supplementary Figure 1 Antioxidant enzyme activities and malondialdehyde content in different treatment groups.** Different lowercase letters showing significant difference at p=0.05 using LSD test. M10 (10 mL/L) (root exudates of watermelon inoculated with *F. mosseae*); M5 (5 mL/L) (the same as M10); V10 (10 mL/L) (root exudates of watermelon inoculated with *G. versiforme*); V5 (5 mL/L) (the same as V10); C10 (10 mL/L) (root exudates of watermelon inoculated with sterilized inoculant); C5 (5 mL/L) (the same as C10); W (equivalent distilled water)

**
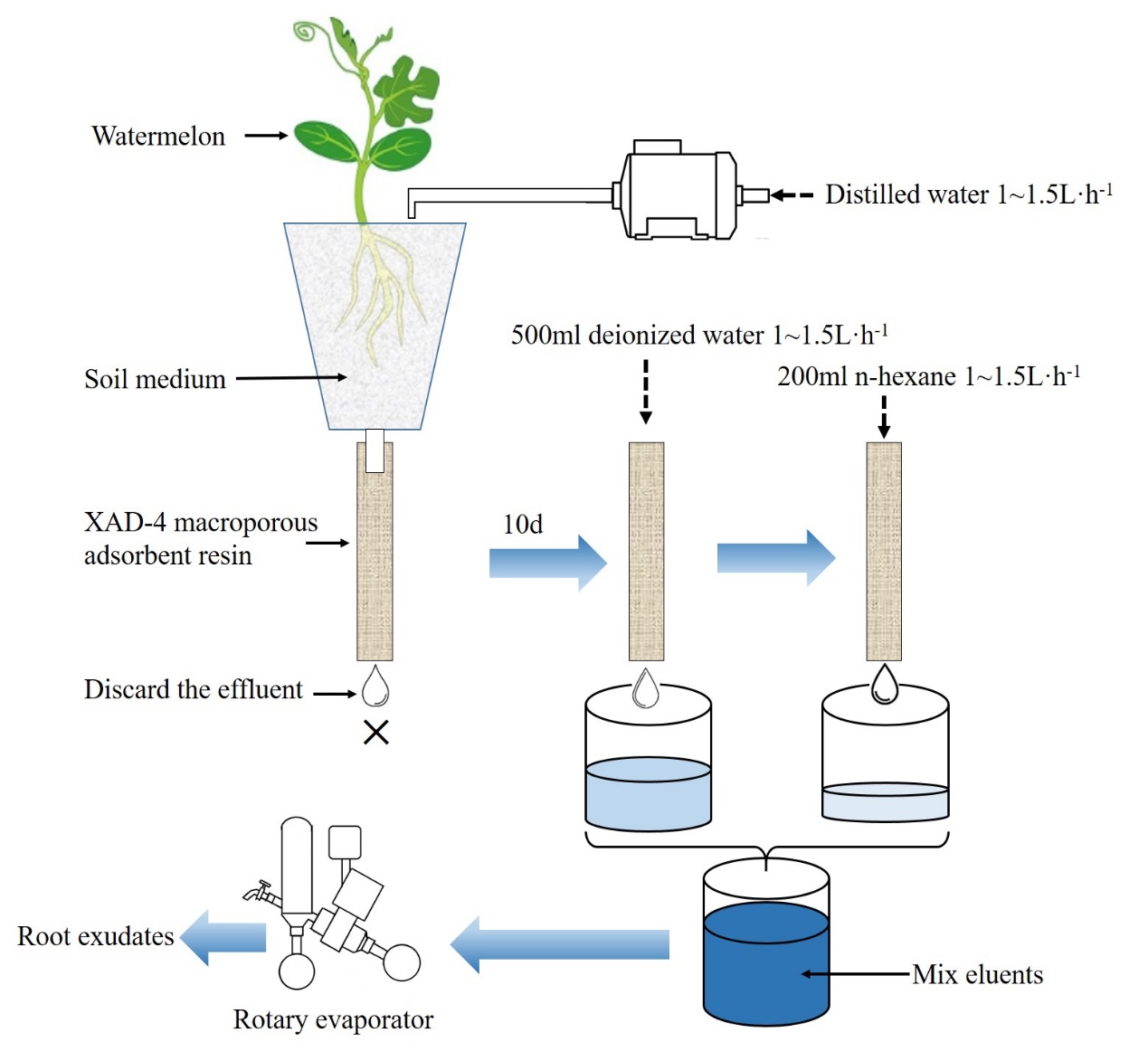
**

**Supplementary Figure 2 Sketch map of the collection process of root exudates**
